# Fabrication of Stacked Graphene Oxide Nanosheet Membranes Using Triethanolamine as a Crosslinker and Mild Reducing Agent for Water Treatment

**DOI:** 10.3390/membranes8040130

**Published:** 2018-12-13

**Authors:** Keizo Nakagawa, Shintaro Araya, Misato Kunimatsu, Tomohisa Yoshioka, Takuji Shintani, Eiji Kamio, Hideto Matsuyama

**Affiliations:** 1 Center for Membrane and Film Technology, Graduate School of Science, Technology and Innovation, Kobe University, 1-1 Rokkodai, Nada, Kobe 657-8501, Japan; 171p101p@stu.kobe-u.ac.jp (S.A.); tom@opal.kobe-u.ac.jp (T.Y.); shintani@port.kobe-u.ac.jp (T.S.); 2 Center for Membrane and Film Technology, Department of Chemical Science and Engineering, Kobe University, 1-1 Rokkodai, Nada, Kobe 657-8501, Japan; 184t420t@stu.kobe-u.ac.jp (M.K.); e-kamio@people.kobe-u.ac.jp (E.K.)

**Keywords:** stacked nanosheet membrane, graphene oxide, water treatment, triethanolamine, reduction, structural stability

## Abstract

Two-dimensional (2D) nanosheets show promise for the development of water treatment membranes with extraordinary separation properties and the advantages of atomic thickness with micrometer-sized lateral dimensions. Stacked graphene oxide (GO)-based membranes can demonstrate unique molecular sieving properties with fast water permeation. However, improvements to the structural stability of the membranes in water to avoid problems such as swelling, disruption of the ordered GO layer and decreased rejection are crucial issues. This study reports the fabrication of stacked GO nanosheet membranes by simple vacuum filtration using triethanolamine (TEOA) as a crosslinker and mild reducing agent for improved structural stability and membrane performance. Results show that GO membranes modified with TEOA (GO-TEOA membranes) have a higher structural stability in water than unmodified GO membranes, resulting in improved salt rejection performance. Furthermore, GO-TEOA membranes show stable water permeance at applied pressures up to 9 bar with Na_2_SO_4_ rejection of 85%, suggesting the potential benefits for water treatment applications.

## 1. Introduction

Membrane separation has become an advanced technology for solving environmental and energy issues. Nanomaterials for membranes have been extensively studied to overcome the limitations of conventional polymeric or inorganic membrane materials, particularly for water treatment due to the high demand for clean water at low cost [1]. Two-dimensional (2D) nanosheets show promise for the development of membranes with extraordinary separation properties and the benefits of atomic thickness with micrometer-sized lateral dimensions. Common materials for nanosheets include graphene-based materials [2,3,4,5,6,7,8,9,10,11,12,13], transition metal dichalcogenides [14,15,16,17] and transition metal oxides [18,19]. Among these, GO (graphene oxide), a derivative of graphite and GO-based membranes have been extensively studied because their unique molecular sieving properties with fast water permeation, simple fabrication method as well as surface hydrophilic properties are attractive for water treatment. GO contains many oxygen functional groups, such as hydroxyl, carboxyl and epoxy groups, that reside on the basal plane and at the edges [20]. Stacked GO membranes are formed by assembling nanosheets into ultrathin membranes via various processes including vacuum filtration [3,5,7,8], pressure-assisted filtration [9], the layer-by-layer approach [10,13] and the wet phase inversion method [12]. The nanochannels between the stacked GO nanosheets allow for fast water permeation and selective rejection of various ions and solutes.

Improving the structural stability of stacked GO nanosheet membranes to reduce issues such as swelling and disruption of the ordered GO layer is an important issue because negatively-charged GO nanosheets easily delaminate in water due to electrostatic repulsion [21,22,23,24]. Decreased water permeation at high applied pressure due to structural changes has also been reported [3]. Recent studies have revealed that the stability of GO nanosheet membranes can be improved with a surface reduction treatment of GO and crosslinking between GO layers. Various methods for the reduction treatment of the GO surface such as thermal treatment [25] and chemical reduction using hydrofluoric acid [26] and hydroiodic acid [21] have been reported. However, for the reduced GO (rGO) membranes, water permeation is greatly decreased because the nanochannel size is narrower and the surface hydrophobicity increases. On the other hand, crosslinking techniques using multivalent cations [22] and chemical binders [13,27] have also been suggested. The technique using multivalent cations could improve the mechanical strength; however, interlayer metal cations were easily left out from GO nanosheet membranes due to ionic exchange with hydrochloric acid or other monovalent cations, leading to disintegration [22]. Hu M. et al., examined GO membranes made via the layer-by-layer approach and cross-linked by 1,3,5-benzenetricarbonyl trichloride (TMC). The GO membrane was stable due to the cross-linking by TMC and showed high water flux but low rejection of monovalent and divalent salts [13]. Thebo K. H. et al. reported that a cross-linked rGO membrane using tannic acid and theanine amino acid as reducing agents and chemical binders that expand a narrow interlayer of rGO showed superior water permeation and structural stability [27]. However, the GO membranes using a crosslinking agent with such large molecular size could not efficiently separate salts and solutes with lower molecular weights. To fabricate GO nanosheet membranes which are stable and demonstrate good separation performance, it is necessary to determine an appropriate crosslinking agent.

Because of its chemical binding and mild reducing abilities for GO, as well as its relatively small molecular size, this study uses triethanolamine (TEOA) as a modifier to fabricate stacked GO nanosheet membranes by simple vacuum filtration for the improvement of membrane performance and stability. The structural stability of the cross-linked GO membranes was also examined under different pressure conditions.

## 2. Materials and Methods

### 2.1. Materials

All chemical regents used in this study were of analytical grade with over 99% purity and were obtained from Wako Pure Chemical Industries, Osaka, Japan unless otherwise stated. Milli-Q water (>18.2 MΩ·cm, Merck Millipore, Billerica, MA, USA) was used for the preparation of all aqueous solutions.

### 2.2. GO Nanosheet Synthesis

GO nanosheets were synthesized by an improved Hummers’ method [28,29]. For this method, a concentrated H_2_SO_4_/H_3_PO_4_ (360:40 mL) mixed solution was added to a mixture of 3.0 g of graphite and 18.0 g of KMnO_4_. The mixed solution was stirred at 50 °C for 12 h, then cooled to room temperature and poured onto 400 mL of ice with 3 ml of 30% H_2_O_2_. After the mixed solution was stirred for another 30 min, it was centrifuged at 1000 rpm for 5 min and the precipitates were removed. This process was repeated several times until no obvious precipitate was left after centrifugation. Then the suspension was centrifuged at 8000 rpm for 15 min and the supernatant was decanted away. The solid material remaining was washed successively with 200 mL of water, 200 mL of 30% HCl and 200 mL of ethanol. After that, the residual material was re-dispersed in Milli-Q water to prepare a 5.0 mg/L GO dispersion.

### 2.3. Fabrication of Stacked GO Nanosheet Membranes

Stacked GO membranes were fabricated by simple vacuum filtration on a surface-modified porous cellulose nitrate support (Millipore Corp.) using a colloidal solution of graphene oxide nanosheets. The cellulose nitrate support (pore size: 50 nm) was immersed in a solution containing 2.5 vol % 3-aminopropyl-triethoxysilane (APTES; Shin-Etsu Chemical Co., Tokyo, Japan) for 2 h, then removed and immersed in Milli-Q water for another 2 h. The modified support was washed with 100 mL of Milli-Q water using a vacuum filtration system and subsequently used for the fabrication of stacked GO nanosheet membranes. The membrane was assembled by vacuum filtration of a GO nanosheet colloidal solution with TEOA. The TEOA was added to the GO nanosheet colloidal solution and sonicated for 0.5 h. Weight ratios of TEOA to GO (TEOA/GO) were prepared at 0.5, 1 and 2 by changing the amount of TEOA. A 50 mL volume of 5.0 mg/L colloidal solution of GO nanosheet including TEOA yielded a membrane with a thickness of approximately 100 nm. The GO membrane thickness was controlled by adjusting the volume of GO colloidal solution for the vacuum filtration. The obtained membranes were vacuum dried for two hours to remove water and strengthen the adhesion between GO nanosheets. The stacked GO nanosheet membranes modified with TEOA are denoted as GO-TEOA membranes. For comparison, the rGO membrane was fabricated by thermal treatment of the GO membrane without any crosslinkers at 70 °C for 72 h in an air atmosphere.

### 2.4. Membrane Characterization

Transmission electron microscope (TEM) images of the fabricated membranes were observed using a JEM-2100F electron microscope (JEOL Ltd., Tokyo, Japan). Field Emission Scanning Electron Microscope (FE-SEM) images were observed using JSF-7500F electron microscope (JEOL Ltd.). Atomic force microscopy (AFM) images were observed using a SPA-400 (Hitachi High-Tech Science, Tokyo, Japan). The AFM observation was performed with an OMCL-AC160TS-C3 cantilever (OLYMPUS, Tokyo, Japan) in dynamic force mode. The crystal structures of the fabricated membranes were measured by powder X-ray diffraction (XRD) (Ultima IV Protectus, Rigaku Corp., Tokyo, Japan) using monochromatized Cu Kα radiation (at 40 kV and 40 mA). The ζ-potential of the sample surfaces was measured using an electrokinetic analyzer (SurPASS™ 3; Anton Paar, Graz, Austria) in 1 mmol/L of KCl aqueous solution. The surface chemical state of the membrane was analyzed using XPS (JPS-9200, JEOL Ltd.). Raman spectroscopy was recorded using a 532 nm laser (NRS-7100, JASCO, Tokyo, Japan). The samples for Raman spectroscopy were prepared by dropping each colloidal solution on a glass plate and drying.

### 2.5. Membrane Performance Evaluation

Membrane performance was evaluated with a cross-flow membrane filtration system [19] consisting of a membrane cell, pump (PCS Pump, SP-22-32-P; FLOM, Inc., Tokyo, Japan) to control the feed water flow rate, back pressure valve for the control of applied pressure and reservoirs of feed, permeate and condensate solutions. The applied pressure and the flow rate of feed water were fixed at 0.2 MPa and 2.0 mL/min, respectively. To evaluate pressure-dependent water flux, the applied pressure was adjusted from 1 to 9 bar. The feed water side of the cell was continuously stirred using a magnetic stirrer. The membrane effective area was 7.07 × 10^−4^ m^2^. Water permeance was calculated from the mass increase of permeate solution. All data were collected from a minimum of three independently-fabricated membranes and averaged for each experimental condition. The rejection of anionic dyes, Acid red 265 (AR; Tokyo Chemical Industries, Tokyo, Japan, Mw: 635.6) and Evans blue (EB; Mw: 960.8) and salts (NaCl and Na_2_SO_4_) were investigated. Aqueous solutions containing 10 ppm of MO, AR and EB were used as the feed solutions. The concentrations of MO, AR and EB in solution were estimated by measuring the absorbance, which was maximized at around λ = 463 nm for MO, 542 nm for AR and 609 nm for EB, with a UV/Vis spectrophotometer (V-650, JASCO International Co., Ltd.). For the measurement of salt rejection, aqueous solutions of 500 ppm each NaCl and Na_2_SO_4_ were used as feed waters. The concentrations of NaCl and Na_2_SO_4_ in the permeate were analyzed using a conductivity meter (LAQUAtwin B-722; HORIBA, Ltd., Kyoto, Japan). The rejection of each solute was calculated as follows: *R* (%) = (1 − *C_P_*/*C_F_*) × 100, where *R* is the rejection and *C_F_* and *C_P_* are the concentrations of the feed and the permeate solutions, respectively.

## 3. Results and Discussion

### 3.1. Fabrication of Stacked GO Nanosheets Membranes Modified with TEOA

**Figure 1:**
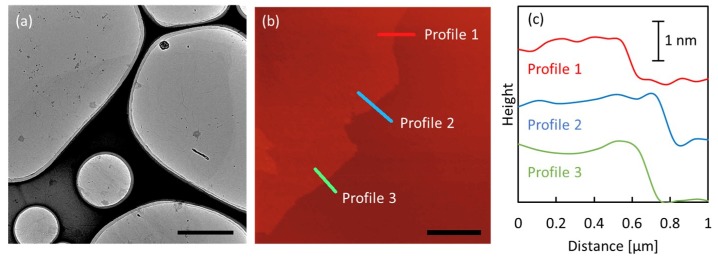
(**a**) Transmission electron microscope (TEM) image of graphene oxide (GO) nanosheets; (**b**) atomic force microscopy (AFM) image and (**c**) the corresponding depth profiles of GO nanosheets. Scale bar: (**a**) 2 µm; (**b**) 2 µm.

Figure 1 shows TEM and AFM images of the prepared GO nanosheets. These images show an exfoliated GO thin layer with a large lateral size (over 5 μm). As shown in Figure 1c, the depth profiles obtained by analyzing the AFM image demonstrate that the heights of GO nanosheets range from 1–1.5 nm, indicating that the GO nanosheets consist of single and double layers.

**Figure 2:**
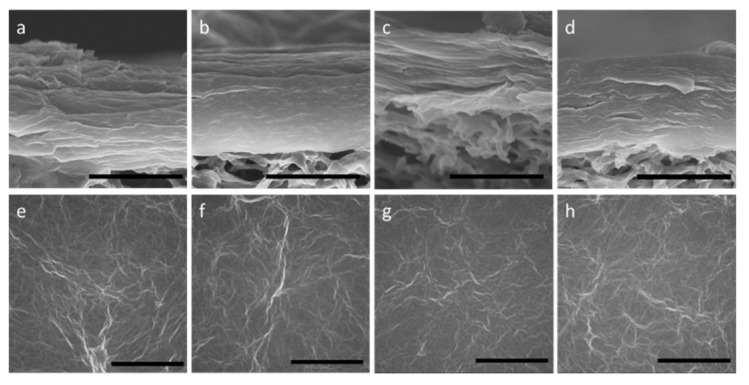
SEM images of cross-sections and overviews of the unmodified GO membrane (**a**) and (**e**) and GO membranes modified with TEOA (GO-TEOA membranes) fabricated with TEOA/GO = 0.5 (**b**) and (**f**); 1 (**c**) and (**g**) and 2 (**d**) and (**h**). Scale bar: 1 µm in (**a**–**d**); 10 µm in (**e**–**h**).

Figure 2 shows SEM images of cross-sections and overviews of the unmodified GO membrane and GO-TEOA membranes fabricated with different TEOA/GO. The cross-section images show that the GO nanosheets were stacked on the support and the total thickness was approximately 1 μm for all membranes. GO nanosheets seem to be interconnected to each other and form a thin layer on the modified cellulose nitrate support. The overview SEM images show rough surfaces with many wrinkles for all membranes; these wrinkles are intrinsic to graphene with a flexible structure. It was observed that the addition of TEOA into GO membranes had little effect on the membrane surface and layered structures. Even when the membrane was as thin as 100 nm, the morphology of the cross-section and surface was similar to those of the thicker GO membranes.

**Figure 3:**
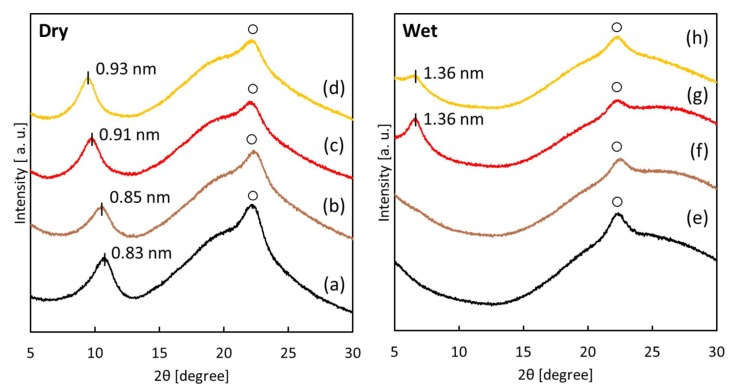
X-ray diffraction (XRD) patterns of the unmodified GO membranes (**a**) and (**e**) and GO-TEOA membranes fabricated with TEOA/GO = 0.5 (**b**) and (**f**); 1 (**c**) and (**g**) and 2 (**d**) and (**h**) in dry and wet conditions. Note that “◦” represents the cellulose nitrate support.

Figure 3 shows XRD patterns of the unmodified GO membrane and GO-TEOA membranes fabricated with different TEOA/GO ratios in dry and wet conditions. In dry conditions (Figure 3a–d), diffraction peaks of the (010) plane derived from the interlayer spacing of the stacked GO membranes were observed at around 2*θ* = 9–12°. For the GO membrane, an interlayer spacing between adjacent GO nanosheets was 0.83 nm. Larger interlayer spacing was obtained for the GO-TEOA membranes when compared with the unmodified GO membrane and the interlayer spacing increased with increasing TEOA/GO value. It is assumed that the differences in the interlayer spacing are due to the existence of TEOA molecules between the GO nanosheets. On the other hand, when we investigated the structural differences by XRD in dry and wet conditions, drastic changes were observed for GO membranes following immersion (Figure 3e–h). It was found that peaks were not observed for the unmodified GO membrane and GO-TEOA membrane fabricated with TEOA/GO = 0.5, whereas a small peak with an interlayer spacing of 1.36 nm was observed for each GO-TEOA membranes fabricated with TEOA/GO = 1 and 2. We assume that the reason for the missing peaks was due to an enlargement of the interlayer distance because of the severe swelling of the layered structure in water and/or disruption of the alignment of stacked GO layer. This phenomenon has been previously reported [21,22,23,24] and occurs because of the highly hydrophilic functional groups on GO nanosheets. These results clearly indicate that GO-TEOA membranes fabricated with TEOA/GO > 1 retain higher structural stability in water than the unmodified GO membrane, although the peak shifted to a lower angle when compared with dry conditions.

**Figure 4:**
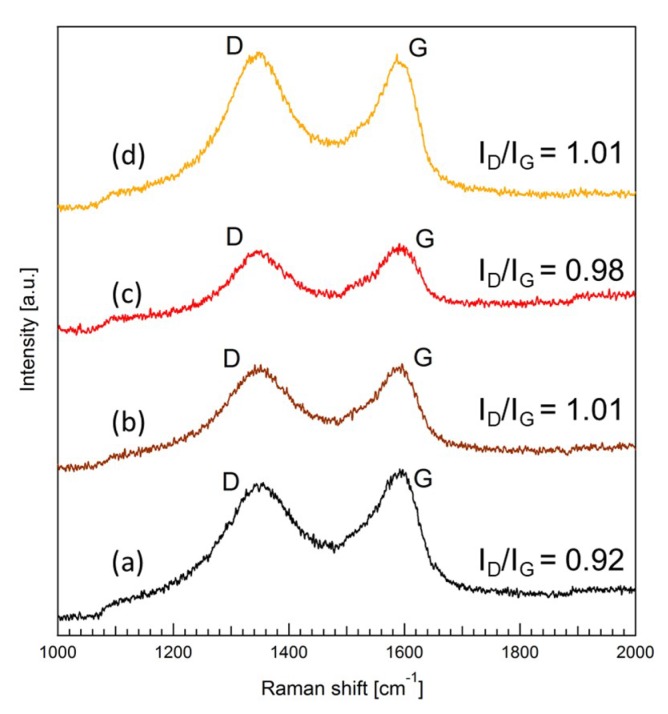
Raman spectra of (**a**) GO and GO-TEOA samples prepared with TEOA/GO = 0.5; (**b**) 1; (**c**) and 2 (**d**).

**Figure 5:**
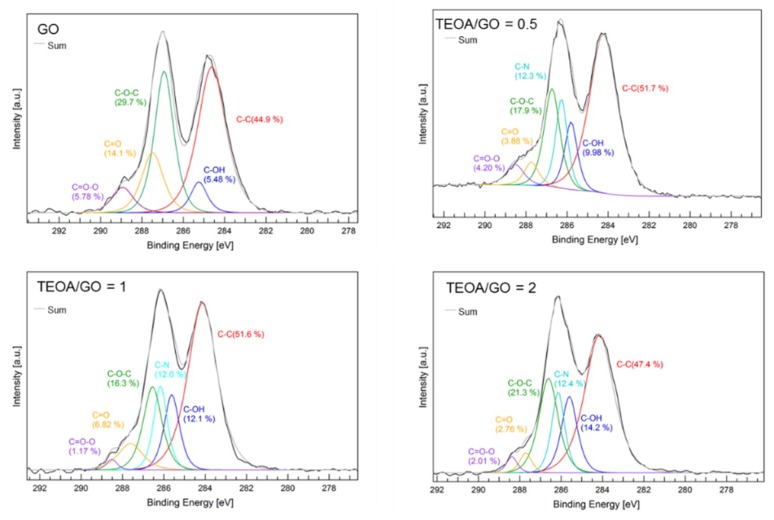
C1s XPS spectra of a GO membrane and GO-TEOA membranes fabricated with different TEOA/GO.

Structural changes of the GO were monitored by Raman and XPS measurements. Figure 4 shows Raman spectra of GO and GO-TEOA nanosheet samples prepared with different TEOA/GO. Two peaks at 1345–1355 cm^−1^ and at 1585–1600 cm^−1^ were observed for the GO sample. These peaks are identified as D and G peaks that are attributable to vibrations of sp^2^ hybridized carbon domains [30,31]. The D peak is originated in out-of-plane vibrations of sp^2^ hybridized carbon domains which result from structural defects and the G peak comes from in-plane vibrations of sp^2^ hybridized carbon domains. A sensitive evaluation of the disorder and defects that exist on graphene structure can be obtained by the intensity ratio of the D peak to G peak (*I_D_*/*I_G_*) [30,31]. It was found that the value of I_D_/I_G_ increased from 0.92 to 1.01 with increasing TEOA/GO ratio. This result implies that the average size of the sp^2^ domains decreased upon reduction of the exfoliated GO [32]. The value of *I_D_*/*I_G_* of all GO-TEOA samples remained at approximately 1.0 and did not change with TEOA/GO ratio. Figure 5 shows C1s XPS spectra of the unmodified GO membrane and GO-TEOA membranes fabricated with different TEOA/GO ratios and Table 1 shows the relative atomic percentage of the chemical binding groups. For the unmodified GO membrane, the huge peak of C-C at 284.6 eV and C-O-C at 286.9 eV were observed. The whole spectra can be divided into 5 peaks: C-OH, C-O-C, C=O, C=O-O and C-C. On the other hand, the huge peaks of C-C and C-O-C were observed at 285.0–287.5 eV for GO-TEOA membranes. The entire spectra consists of 6 peaks of C-N for GO modified with TEOA and C-OH, C-O-C, C=O, C=O-O and C-C for GO [33]. For all GO-TEOA membranes, it was found that the ratio of C-C, C-OH and C-N increased but that of C-O-C, C=O and C=O-O decreased compared with the unmodified GO membrane. These Raman and XPS spectra indicate that the oxygen-containing functional groups in GO-TEOA membranes were slightly reduced. Liu G. et al., reported that TEOA has a mild reducing ability as a chemical modifier [33]. The N^+^ groups formed by hydrolysis of TEOA are used for ring-opening reactions of epoxides, which leads to the formation of C=C bonds. The changes of the ratio of the chemical binding group are consistent with this report. The ratio of C-OH apparently increased with increasing TEOA/GO ratio, suggesting the incorporation of TEOA between GO nanosheets.

**Figure 6:**
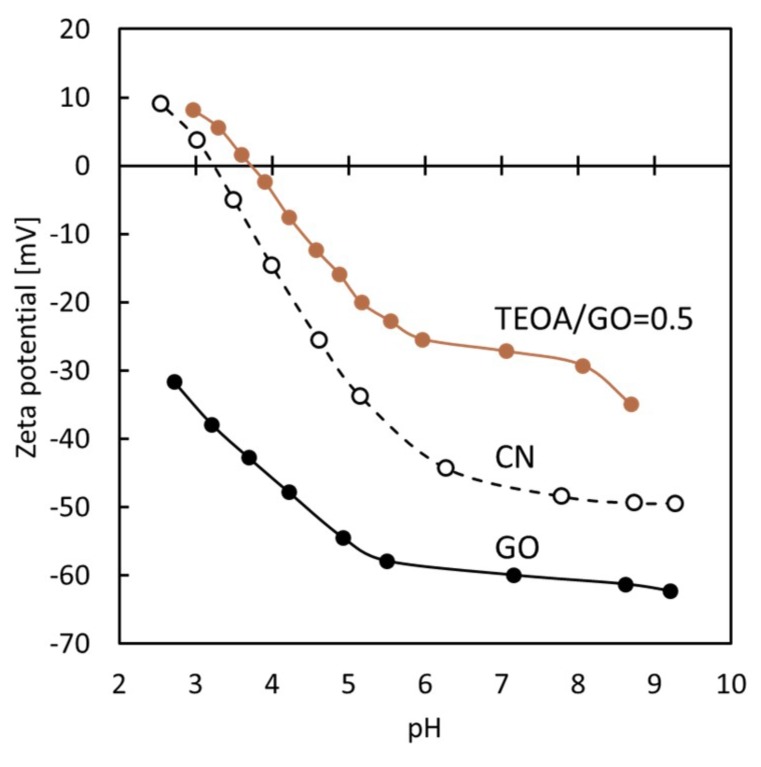
Zeta potential of the GO membrane, GO-TEOA membranes fabricated with TEOA/GO = 0.5 and the cellulose nitrate support membrane.

In streaming potential measurements, the surface charge (ζ-potential) of the unmodified GO membranes, GO-TEOA membranes fabricated with TEOA/GO = 0.5 as well as the cellulose nitrate support membrane were measured at several pH values (Figure 6). The results reveal that the unmodified GO membrane was negatively charged across a wide pH range. In contrast, for the GO-TEOA membrane fabricated with TEOA/GO = 0.5, the ζ-potential was weakened by the introduction of TEOA and found to be dependent on pH, with an iso-electric point of approximately 3.7. It has been reported that N^+^ groups in TEOA form quaternary nitrogen species with carboxyl groups on GO surfaces via electrostatic interaction [33]. Thus, for GO-TEOA membranes, it is assumed that TEOA molecules were intercalated into the interlayers via electrostatic interaction, resulting in the attenuation of the negative charge of GO nanosheets.

It is interesting to note that the GO-TEOA membranes had high structural stability in water when compared with the unmodified GO membrane. This stability comes from the role of the TEOA as a chemical binder. The XPS analysis indicates that TEOA molecules exist in the fabricated GO nanosheet membranes. During the formation of quaternary nitrogen species, hydroxyl groups in TEOA can be introduced onto the GO [33]. Thus, TEOA can crosslink the GO nanosheet layers effectively via electrostatic interactions between N^+^ groups in TEOA and carboxyl groups on GO and via hydrogen bonding between hydroxyl groups of TEOA, that strengthen the final membrane. In addition, the attenuation of the negative charge of GO nanosheets would inhibit the electrostatic repulsion between GO nanosheets in water, leading to an enhancement of membrane structural stability.

### 3.2. Membrane Performance

**Figure 7:**
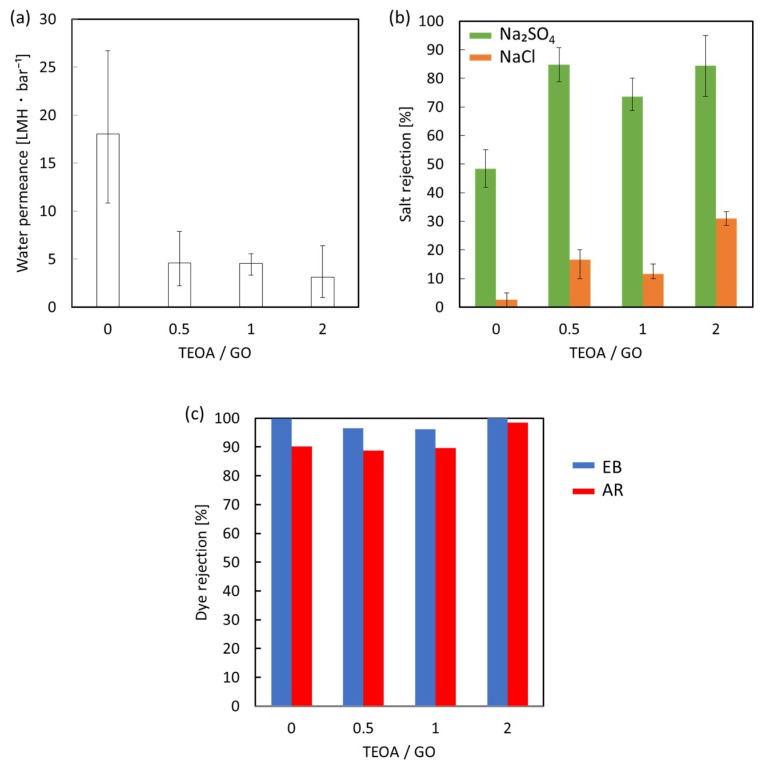
(**a**) Water permeance, (**b**) salt rejection and (**c**) rejection of EB and AR by the unmodified GO membrane and GO-TEOA membranes fabricated with different TEOA/GO ratios.

Figure 7 shows the water permeance and the rejection of salts and organic dyes for the unmodified GO membrane and GO-TEOA membranes fabricated with different TEOA/GO ratios. The performance tests were conducted with the cross-flow membrane system. A higher water permeance was observed for the unmodified GO membranes (18 LMH·bar^−1^) when compared with the GO-TEOA membranes (3–7 LMH·bar^−1^). However, it should be noted that the unmodified GO membrane and the GO-TEOA membranes fabricated with TEOA/GO = 0.5 had inconsistent water permeance due to structural instability in water, as indicated with XRD results. Membrane rejection performance was evaluated using salts (Na_2_SO_4_ and NaCl) and anionic dyes (EB and AR). The unmodified GO membrane showed rejection of less than 3% for NaCl and approximately 50% for Na_2_SO_4_. In contrast, the GO-TEOA membranes showed higher rejections of 10–30% for NaCl and 70–85% for Na_2_SO_4_. Although the membrane performance results for the unmodified GO membrane and the GO-TEOA membranes fabricated with TEOA/GO = 0.5 include some inconsistencies, in general, the trend observed was that increasing the TEOA/GO ratio increased salt rejection and decreased water permeance. The unmodified GO membrane and the GO-TEOA membranes demonstrated superior rejection performances for organic dyes: greater than 95% for EB and greater than 90% for AR. Table S1 summarizes membrane performance of the GO-TEOA membrane (TEOA/GO = 2), GO membranes in the literature and commercial nanofiltration (NF) membranes. The GO-TEOA membrane exhibited comparable rejection performance to the GO membranes and the commercial polymer-based NF membranes but lower permeance. The separation performance for GO membranes can be explained by molecular sieving between sheets and electrostatic repulsion (Donnan exclusion mechanism [34,35]) between negatively charged sheets and anionic species. XRD results indicate that the GO-TEOA membranes show stable interlayer spacing of 1.36 nm while the unmodified GO membrane would have larger or disordered channel structures. Although the surface negative charge was attenuated by the reaction between GO and TEOA for the GO-TEOA membranes, the electrostatic repulsion of anions could be expected in the 2D nanochannels. These factors may explain why lower water permeance but higher rejection of salts were obtained for GO-TEOA membranes.

**Figure 8:**
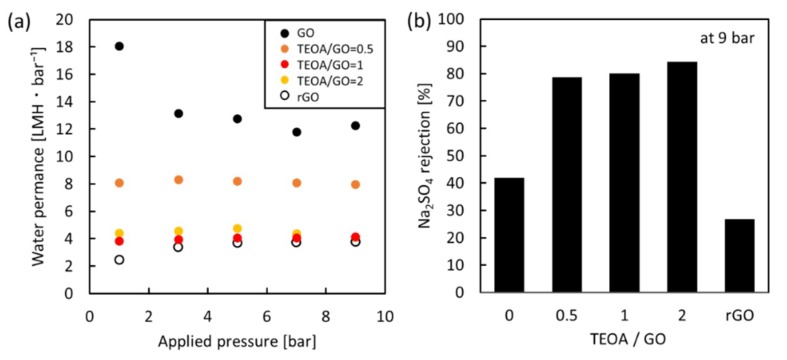
(**a**) Pressure-dependent water permeance of the unmodified GO membranes, GO-TEOA membrane fabricated with different TEOA/GO ratios and the rGO membrane; (**b**) Na_2_SO_4_ rejection of each membranes at 9 bar.

Pressure-dependent water permeance was also investigated to confirm the stability of the nanochannels of the GO membranes, given the importance of membrane durability in practice. The water permeance of the unmodified GO membranes and GO-TEOA membranes fabricated with different TEOA/GO ratios under pressures from 1 to 9 bar is shown in Figure 8a. For GO-TEOA membranes, water permeance was stable at approximately 4–8 LMH·bar^−1^ at applied pressures up to 9 bar with Na_2_SO_4_ rejection of 78–84%, suggesting that the nanochannels were rigid enough to withstand external pressures up to 9 bar. Based on typical viscous flow in accordance with the Hagen–Poiseuille equation [5], water flux is proportional to the differential pressure; in other words, water permeance is constant at the differential pressure. The result indicates that the channel structure in the GO-TEOA membranes is maintained under an applied pressure up to 9 bar. On the other hand, for the unmodified GO membrane, water permeance drastically decreased from 18 to 12 LMH·bar^−1^ when the applied pressure exceeded 3 bar. It has been reported that the nanochannels in GO membranes cannot sustain their spacing and begin to collapse, leading to shrinkage, with increasing pressure [3]. Thus, the decrease in water permeance implies that a geometrical change of the nanochannels occurred under pressure loading beyond 3 bar. Furthermore, the pressure-dependent water permeance was compared with the rGO membrane. The results of the characterization are listed in the supplementary information. The rGO membrane also showed stable water permeance (4 LMH·bar^−1^) at applied pressures up to 9 bar. However, Na_2_SO_4_ rejection was very low (less than 30%). This is likely a result of the severe reduction of surface functional groups, leading to a decreasing effect of electrostatic repulsion. Therefore, the pressure loading experiments demonstrated the potential durability of the GO-TEOA membranes.

## 4. Conclusions

GO nanosheet membranes were fabricated with triethanolamine (TEOA) using simple vacuum filtration. XRD results indicated that GO-TEOA membranes had higher structural stability in water compared with the unmodified GO membrane. TEOA plays important roles as a crosslinker between GO nanosheets and as a mild reducing agent to the surface of GO nanosheets. As a result, GO-TEOA membranes demonstrated higher rejection performance for salts than the unmodified GO membranes. In addition, GO-TEOA membranes demonstrated superior rejection performance for two organic dyes. In a pressure loading experiment, the GO-TEOA membrane showed stable water permeance under applied pressure up to 9 bar with Na_2_SO_4_ rejection of 85%. In contrast, the reduced GO membrane showed stable water permeance but low Na_2_SO_4_ rejection due to decreased electrostatic repulsion as a result of the severe reduction of surface functional groups. These findings provide useful information for the design of robust nanochannels in stacked 2D nanosheet membranes. Further investigations are required to precisely control the nanochannel size for enhanced ion and molecular separation.

## Figures and Tables

**Table 1:** The relative atomic percentages of the chemical groups in GO and GO-TEOA membranes.

TEOA/GO	C-C/%	C-OH/%	C-N/%	C-O-C/%	C=O/%	C=O-O/%
0	44.9	5.48	0	29.7	14.1	5.78
0.5	51.7	10.0	12.3	17.9	3.88	4.20
1	51.6	12.1	12.0	16.3	6.82	1.17
2	47.4	14.2 ^1^	12.4	21.3	2.76	2.01

## Supplementary Materials

The following are available online at http://www.mdpi.com/2077-0375/8/4/130/s1, Figure S1: (a) Cross-section and (b) Surface SEM images of the rGO membrane. Scale bar: (a) 1 µm; (b) 10 µm. Figure S2: XRD patterns of the rGO membrane in dry and wet conditions; “◦” denotes the cellulose nitrate support. Figure S3: Raman spectra of the rGO membrane. Figure S4: C 1 s XPS spectra of the rGO membrane.
