# Supplementary material for: Fabrication of Stacked Graphene Oxide Nanosheet Membranes Using Triethanolamine as a Crosslinker and Mild Reducing Agent for Water Treatment
Source: Membranes (Basel). 2018 Dec 13;8(4):130. doi: 10.3390/membranes8040130 (PMC6315452; doi:10.3390/membranes8040130)
Supplement: Supplementary file 1 [file membranes-08-00130-s001.pdf]

Supplementary Materials: Fabrication of Stacked Graphene Oxide Nanosheet Membranes Using Triethanolamine as a Crosslinker and Mild Reducing Agent for Water Treatment

Keizo Nakagawa, Shintaro Araya, Misato Kunimatsu, Tomohisa Yoshioka, Takuji Shintani, Eiji Kamio and Hideto Matsuyama

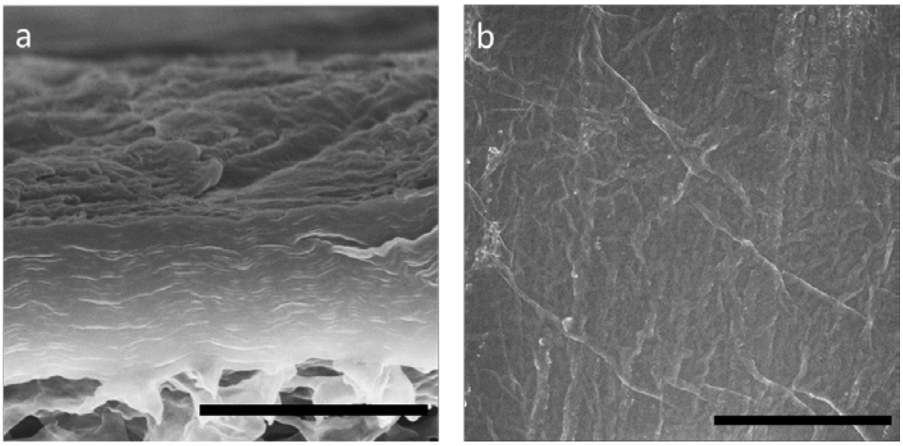

Figure S1. (a) Cross-section and (b) surface SEM images of the rGO membrane. Scale bar: (a) 1 μm, (b) 10 μm.

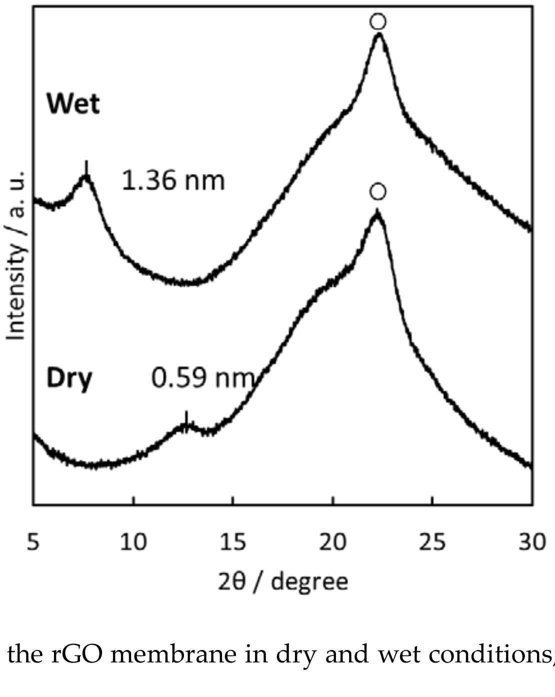

Figure S2. XRD patterns of the rGO membrane in dry and wet conditions; "○" denotes the cellulose nitrate support.

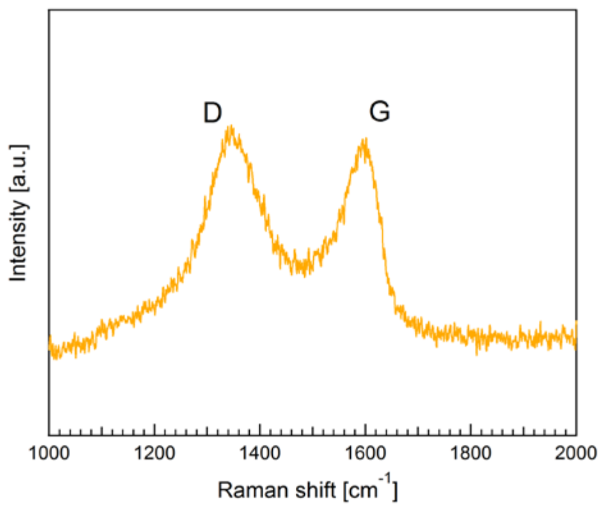

Figure S3. Raman spectra of rGO sample.

The value of I_D/I_G was 1.04, which is larger than those of GO and GO-TEOA samples. This result indicates that the degree of surface reduction of GO modified with TEOA was slightly lower than that of rGO.

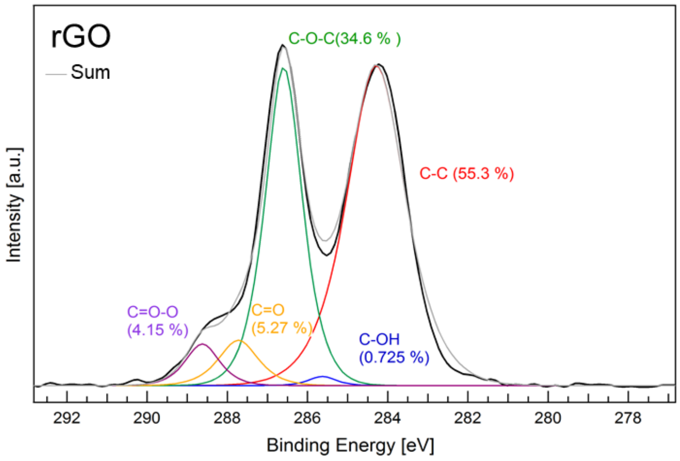

Figure S4. C 1s XPS spectra of the rGO membrane.

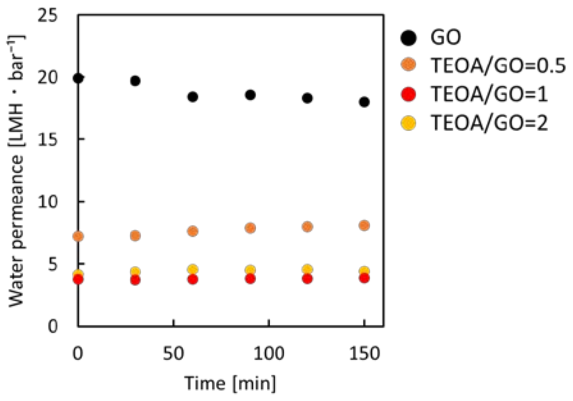

Figure S5. The change of water permeance with time of the unmodified GO membrane and GO-TEOA membranes fabricated with different TEOA/GO ratios at 1 bar.

Table S1. Comparison of performance of GO-TEOA membranes with those of various GO-based membranes and some commercial NF membranes.

Membrane (GO-modifier/support)	Fabrication Method	Thickness (nm)	Permeance (L/m^2 h bar)	Rejection (%) NaCl	Na_2SO_4	Dyes	Reference
GO-TEOA/ cellulose nitrate	Vacuum filtration	100	3	31	84	EB 100 AR 98.4	This work
GO-TMC/polydopamine modified polysulfone	Layer-by-layer	25-30	30	29	26	MB 66	[1]
GO-polyethyleneimine/ polyacrylonitrile	Electric field-assisted layer-by-layer	8	16.4	22.6	86.8	-	[2]
S-rGO-HPEI/nylon	Vacuum filtration	18	85.2	-	-	EB 100 MB 98.6	[3]
NTR-7450 (Nitto Denko)	-	-	9.2	51	92	-	[4]
NTR-7410 (Nitto Denko)	-	-	50.0	15	55	-	[4]

TMC: 1,3,5-benzenetricarbonyltrichloride, S-rGO-HPEI: solvent solvated rGO-hyperbranched poly(ethylene imine), EB: Evans blue (Mw: 960.8), AR: Acid red 265 (Mw: 635.6), MB: Methylene blue (Mw: 319.9).

References

1. Hu, M.; Mi, B. Enabling Graphene Oxide Nanosheets as Water Separation Membranes. *Environ. Sci. Technol.* **2013**, *47*, 3715–3723, doi:10.1021/es400571g.
2. Wang, T.; Lu, J.; Mao, L.; Wang, Z. Electric field assisted layer-by-layer assembly of graphene oxide containing nanofiltration membrane. *J. Memb. Sci.* **2016**, *515*, 125–133, doi:10.1016/j.memsci.2016.05.053.
3. Huang, L.; Chen, J.; Gao, T.; Zhang, M.; Li, Y.; Dai, L.; Qu, L.; Shi, G. Reduced Graphene Oxide Membranes for Ultrafast Organic Solvent Nanofiltration. *Adv. Mater.* **2016**, *28*, 8669–8674, doi:10.1002/adma.201601606.
4. Zhang, W.; He, G.; Gao, P.; Chen, G. Development and characterization of composite nanofiltration membranes and their application in concentration of antibiotics. *Sep. Purif. Technol.* **2003**, *30*, 27–35, doi:10.1016/S1383-5866(02)00095-3.
